# Extraction and concentration of nanoplastic particles from aqueous suspensions using functionalized magnetic nanoparticles and a magnetic flow cell

**DOI:** 10.1186/s43591-022-00051-1

**Published:** 2023-01-27

**Authors:** Mark C. Surette, Denise M. Mitrano, Kim R. Rogers

**Affiliations:** 1 U.S. EPA Center for Environmental Measurement and Modeling, 109 T.W. Alexander Drive, Research Triangle Park, Durham, NC 27709, USA; 2 WSP USA Solutions, Inc, 18300 NE Union Hill Road Suite 200, WA 98052 Redmond, USA; 3 Department of Environmental Systems Science, Institute of Biogeochemistry and Pollutant Dynamics, ETH Zürich, Universitatstrasse 16, 8092 Zürich, Switzerland

## Abstract

Although a considerable knowledge base exists for environmental contamination from nanoscale and colloidal particles, significant knowledge gaps exist regarding the sources, transport, distribution, and effects of microplastic pollution (plastic particles < 5 mm) in the environment. Even less is known regarding nanoplastic pollution (generally considered to be plastic particles < 1 μm). Due to their small size, nanoplastics pose unique challenges and potential risks. We herein report a technique focused on the concentration and measurement of nanoplastics in aqueous systems. Hydrophobically functionalized magnetic nanoparticles (HDTMS-FeNPs) were used as part of a method to separate and concentrate nanoplastics from environmentally relevant matrices, here using metal-doped polystyrene nanoplastics (PAN-Pd@NPs) to enable low-level detection and validation of the separation technique. Using a magnetic separation flow cell, PAN-Pd@NPs were removed from suspensions and captured on regenerated cellulose membranes. Depending on the complexity of solution chemistry, variable extraction rates were possible. PAN-Pd@ NPs were recovered from ultrapure water, synthetic freshwater, synthetic freshwater with a model natural organic matter isolate (NOM; Suwannee River Humic Acid), and from synthetic marine water, with recoveries for PAN-Pd@NPs of 84.9%, 78.9%, 70.4%, and 56.1%, respectively. During the initial method testing, it was found that the addition of NaCl was needed in the ultrapure water, synthetic freshwater and synthetic fresh water with NOM to induce particle aggregation and attachment. These results indicate that magnetic nanoparticles in combination with a flow-through system is a promising technique to extract nanoplastics from aqueous suspensions with various compositions.

## Introduction

Progress has been made in the past decade with respect to elucidating the sources, transport, distribution and effects of nanoscale and colloidal particle pollution. However, while advancements have also been made in macro-, meso-, and microplastic pollution [1–3], numerous knowledge gaps remain that hinder our [4, 5] understanding of the risks posed by nanoplastic pollution. One aspect in particular that is poorly understood is the abundance and hazards posed by nanoplastics (i.e., plastic particles < 1 μm) [5–8]. As summarized by Ter Halle et al. (2017) [9], only ≈1% of the mass of plastic debris that is estimated to enter the global marine environment annually can be accounted for. This has led many researchers to hypothesize that a significant, yet currently unknown, amount of nanoplastic particles may be present [10, 11]. Current evidence suggests that the same physical-chemical processes that have been shown to degrade larger macro- and mesoplastics to smaller microplastics (e.g., mechanical weathering and photo-degradation) likely also generate even smaller nanoplastic particles [12–14]. Considering that the vast majority of plastic pollution originates from upland sources, it is likely that nanoplastics are also present in terrestrial and freshwater environments [1, 15, 16].

The factors hindering the identification of nanoplastics in the environment are due to limitations or deficiencies in either 1) sample collection and processing methods or 2) analytical techniques currently used to identify and quantify small plastic particles [6, 17]. Regarding sampling strategies, samples are typically collected using variously sized sieves or nets, which generally have openings on the order of many micrometers and are thus unable to collect nanoplastics [10, 15]. In addition, while relatively larger micro-to-macroplastics are often physically handled and sorted during sample processing, these techniques cannot be applied to nanoplastics. Lastly, environmental samples that potentially contain nanoplastic particles contain other, non-target biogenic and geogenic particles of comparable size, thus requiring processing steps to reduce matrix complexity and remove non-target particles while preserving the chemical and physical attributes of the nanoplastics. With regards to analytical limitations, the predominant methods used to chemically identify plastic particles are either limited to microplastic particles or larger in size (e.g., μ-Raman spectroscopy, μ-FT-IR) or require a relatively large sample mass (e.g., py-GC-MS). Likewise, optical-based stereomicroscopy techniques commonly used to analyze, characterize, and quantify plastic particles cannot be applied to nanoplastics due to diffraction limits. For these reasons, few studies have offered evidence indicating the presence of nanoplastic particles in the environment [9, 18].

To overcome current analytical limitations, various methods have been proposed including “Raman tweezers” (i.e., laser-based “optical tweezers” coupled to a Raman spectrometer) [19], thermal desorption-proton transfer reaction-mass spectrometry (TD-PTR-MS) [20], and surface-enhanced Raman spectroscopy (SERS) combined with Klarite substrates [21]. Each of these analytical techniques is promising and have demonstrated the ability to chemically identify plastic particles smaller than 1 μm. Collectively, however, they suffer from certain drawbacks including throughput limitations (in some cases limited to single-particle analysis), fairly rigorous and complex data analysis requirements, or only providing semi-quantitative information due to unknown particle physical characteristics (e.g., shape and size) [20]. Nevertheless, to realize the full potential of these advanced analytical techniques, sample collection and handling methods, particularly methods that can effectively isolate and extract nanoplastics from various environmental media, are needed to facilitate their analysis.

To that end, Grbic et al. (2019) [22] recently demonstrated the use of hydrophobically-functionalized magnetic nanoparticles to extract meso- and microplastic particles from environmental samples. Due to the previously mentioned limitations surrounding nanoplastic detection and analysis, their approach was only demonstrated for plastic particles larger than 10 μm. Furthermore, higher variability in sample recovery was observed amongst replicate samples containing particles < 20 μm, which the authors attributed to their experimental procedure and the challenges with accurately quantifying smaller sized particles. Nonetheless, this concept offers advantages relative to the techniques commonly used when sampling plastic particles. First, as a bulk extraction technique, it could theoretically be scaled and applied to large sample volumes (e.g., liters). Second, depending on the experimental set-up, the technique could be modified to simultaneously extract and concentrate plastic particles across various size ranges (e.g., from nano- to microplastics). These two advantages are important when considering that nano- and microplastics are generally expected to have a relatively large size distribution and be present together in the environment, and thus having a technique which can concentrate a wide size range of plastics would expediate characterization of the variously sized plastics in a given sampling campaign [6, 11]. Finally, this method could be utilized as a component within a broader sample processing scheme to enable its application to various environmental media, including soils and sediments.

The objectives of the current research were to demonstrate the use of surface-modified iron nanoparticles to separate and concentrate nanoplastics under laboratory conditions and provide proof-of-concept for a larger, field deployable flow-through system. To circumvent the analytical challenges encountered with detecting and accurately quantifying nanoplastics, polystyrene (PS) nanoplastic particles containing an inorganic (palladium [Pd]) tracer were chosen as model nanoplastics [23]. Using the inorganic tracer embedded within the core of the nanoplastics allowed us to leverage the high sensitivity and low detection limits afforded by inductively coupled plasma-mass spectrometry (ICP-MS) to precisely measure the extraction efficiency of our method under various environmentally relevant conditions. In combination with an in-house built magnetic flow cell, we were able to test the effectiveness of our extraction approach to extend down to nano-sized plastics in aqueous suspensions. Finally, we identified some current limitations of this technique but still highlight how this sample collection and preparation protocol could be applied within a broader environmental sampling campaign to extract plastic particles spanning the nano-to-micro scales.

## Materials and methods

### Metal-doped nanoplastics

Nanoplastics containing an inorganic tracer were chosen as model nanoplastics to overcome current analytical challenges that hinder their detection and quantification in environmental media. Metal-doped nanoplastic particles present a useful proxy for nanoplastic particles found in the environment, and are intended to serve as a tool to support method development [24]. Herein, we utilize nanoplastic particles consisting of a polyacrylonitrile (PAN) core containing a trace-metal label (palladium [Pd]) and a polystyrene (PS) shell (referred to as PAN-Pd@PS NPs). Details regarding the synthesis of the PAN-Pd@PS nanoplastics (NPs) and a demonstration on their utility are provided elsewhere [23, 25]. It is important to note that in the current work, the results are presented and discussed in regard to the measured concentration of Pd, which is proportional to the concentration of nanoplastics present in the sample (0.3% w/w).

Initial characterization of the PAN-Pd@PS NPs was performed using duplicate samples prepared by diluting the stock dispersion to ≈10^10^ particles/mL with ultrapure 18.2 MΩ-cm resistivity water (DDI) and then analyzed via nanoparticle tracking analysis (NTA), dynamic light scattering (DLS), phase analysis light scattering (PALS), and scanning electron microscopy (SEM). The NTA measurements were collected using a NanoSight 500 (Malvern Panalytical) equipped with a green laser (λ = 532 nm), with 5 captures collected per sample (30 seconds/capture). Each capture was collected and processed at the same camera/threshold levels. The DLS and PALS measurements were collected using a ZetaSizer Nano ZS (Malvern Panalytical), with the same samples analyzed via each technique. For the DLS analysis, 3 measurements per sample (6 runs/measurement at 10 seconds/run) were collected while the PALS analysis was performed at 5 measurements per sample (10 cycles per measurement). Finally, SEM micrographs were collected using a Zeiss Σigma VP (Carl Zeiss) operated at an acceleration voltage of 3 kV and a working depth of 5.5 mm while using the InLens secondary electron (SE) detector. Samples were prepared by placing ≈10 μL drop of the dispersion on a carbon-coated nickel grid (Ni-200 mesh; Ted Pella) and then dried overnight in a vacuum desiccator. Once dry, the grids were affixed to an SEM aluminum pin mount using double-sided carbon tabs (Spectro Tabs, Ted Pella). The acquired micrographs were analyzed using the Fiji software package in ImageJ [26, 27].

### Functionalized magnetic nanoparticles

Hydrophobically-functionalized magnetic nanoparticles (HDTMS-FeNPs) were prepared according to the protocol described in Grbic et al. (2019), with some adjustments [22]. Briefly, 19.8 mL of methanol (Sigma-Aldrich; Reagent Grade, 99%) was combined with 0.2 mL of hexadecyltrimethoxysilane (HDTMS; Sigma-Aldrich; Technical Grade > 85%) in a Teflon vial and briefly vortexed before adding ≈40 mg of 25 nm iron nanoparticles (FeNPs; Sigma-Aldrich; Trace Metal Grade, > 99.5%). Upon addition of the FeNPs, the vial was sealed and mixed continuously overnight on a vortex mixer at 480 rpm at room temperature. The HDTMS-FeNPs were not washed with water after mixing but were instead allowed to remain in the HDTMS-methanol mixture (a deviation from the previously published synthesis method). This was done to improve particle shelf-life, as it was found during preliminary tests that HDTMS-FeNPs would slowly aggregate and form a thin film at the airwater interface when removed from the HDTMS-methanol mixture and re-dispersed in ultrapure, 18.2 MΩ-cm resistivity water (DDI).

Duplicate samples of HDTMS-FeNPs were prepared by diluting the stock dispersion to ≈10^10^ particles/mL with DDI and then characterized via the same techniques and conditions as the PAN-Pd@PS NPs. The only exception was the SEM analysis, where the SEM was operated at an acceleration voltage of 20 kV and a working depth of 5.0 mm.

### Experimental setup

**Fig. 1:**
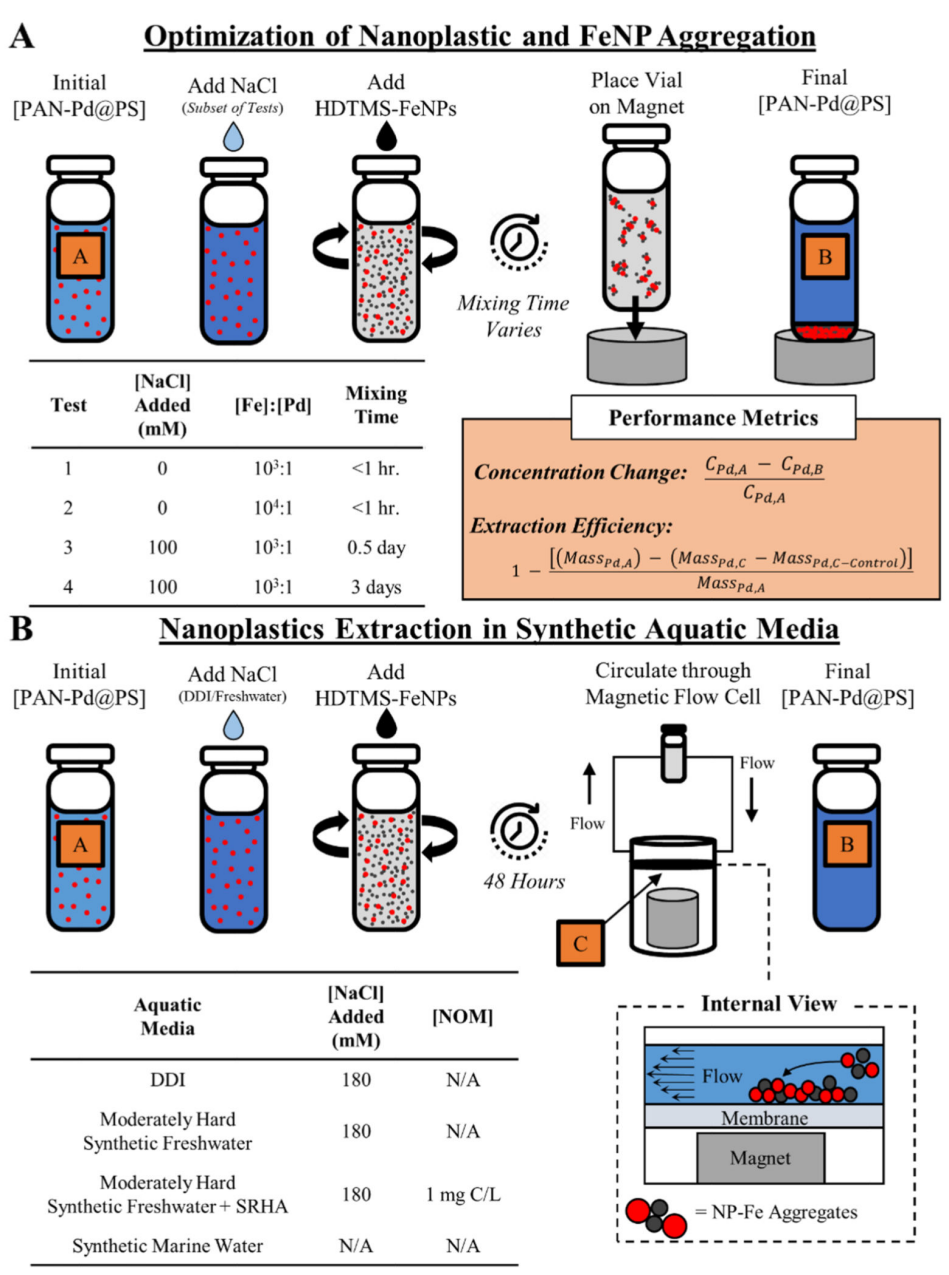
Graphical overview of experimental procedure depicting (**a**) initial optimization method and (**b**) extraction of nanoplastics in synthetic aquatic media using magnetic flow cell system and the optimized conditions. In each panel, the “**A**”, “**B**”, and “**C**” callout boxes correspond to the subscript notation used in the Performance Metrics equations, wherein *C*_*Pd,i*_ indicates the concentration of PAN-Pd@PS NPs measured at the *i*-th step and *Mass*_*Pd,i*_ indicates the corresponding mass of PAN-Pd@PS NPs calculated at the *i*-th step. Samples were used to assess losses of the PAN-Pd@PS NPs via adsorption to the vessel walls and/or tubing within the magnetic flow cell system

A suite of tests were performed to optimize the extraction and concentration method by identifying the mechanism(s) driving interactions between the HDTMS-FeNPs and the PAN-Pd@PS NPs. These diagnostic tests were performed in DDI using a simplified experimental technique to extract the particles from suspension, where the sample vessel was placed on-top of the neodymium magnet for ≈30 minutes before a sub-sample of the supernatant was obtained from the uppermost ≈1 cm layer (Fig. 1a). This experimental procedure allowed for the efficient testing of various experimental conditions that were expected to influence the attachment of the HDTMS-FeNPs to the PAN-Pd@PS NPs, including variations in the concentration of NaCl (0 mM and 100 mM), the mass concentration ratio of [Fe]:[Pd] (10^3^:1 and 10^4^:1), and the mixing time (i.e., the duration of the resting period; between < 1 hour to 3 days) were examined.

Following the optimization tests, experiments were performed using the refined experimental conditions to assess the ability of a magnetic flow cell system to extract and concentrate the nanoplastics in increasingly more complex and environmentally relevant aquatic media (Fig. 1b, Fig. S1). Our intent in using this device was to demonstrate a proof-of-concept approach that could be easily scaled to process larger sample volumes (i.e., towards a field-deployable system) while enabling the physical transport and handling of the extracted nanoplastics for further analysis. For the magnetic flow device, a 10 kDa regenerated cellulose (RC) membrane was placed underneath the flow path within the flow cell enclosure but above the neodymium magnet (Fig. 1b and Fig. S1). In this position, the dispersion flows over the top of the membrane before exiting the channel (i.e., there is no flow through the membrane), where the membrane simply acts as a removable surface that the particles are extracted onto. This affords certain advantages, including the ability to directly image the extracted particles via SEM, analyze a sub-sample of the membrane (collected using a biopsy punch, for example) via py-GC-MS to chemically identify the extracted particles, or to recover the extracted particles by washing the membrane.

To establish the baseline performance of the system under idealized conditions that minimize potential interferences from the sample media, the initial extraction tests used DDI as the dispersion media. Subsequent extraction tests were conducted using synthetic freshwater (U.S. EPA Moderately Hard Water) without and with a model natural organic matter (NOM) surrogate (Suwannee River Humic Acid [SRHA]; 3S101H International Humic Substances Society) at a total organic carbon concentration (TOC) of 1 mg C/L, and synthetic marine water (InstantOcean^®^). Details regarding the preparation of and constituents in the synthetic aquatic media are provided in the Supplementary Information.

For each aquatic media, six replicate 5 mL samples were prepared by combining the requisite volume of 0.2 μm-filtered media (WhatMan Antotop syringe filter) and subsequently adding an aliquot of the stock PAN-Pd@PS NP dispersion in a glass conical vial to a nominal target concentration of 100 μg Pd/L (equivalent to ≈10^12^ particles/L). As is discussed below, the initial optimization tests revealed that the ionic strength of the sample needed to be sufficiently high such that aggregation between the PAN-Pd@PS NPs and HDTMS-FeNPs would occur. Thus, for the media with initially low ionic strength (DDI and synthetic freshwater), a 150 μL aliquot of 0.2 μm-filtered 6 M NaCl was added to raise the sample’s ionic strength (*I*) to ≈180 mM. Following the addition of the PAN-Pd@PS NPs (and NaCl, if needed), a 1 mL sub-sample was collected via pipette and transferred to a 15 mL polypropylene tube (Falcon Fischer Scientific). A 1 mL aliquot of the stock HDTMS-FeNP dispersion was then added to the remaining volume in the sample vessel, targeting a nominal concentration of 200 mg Fe/L and resulting in a mass concentration ratio of [Fe]:[Pd] ≈ 1 0^3^:1 (equivalent to a number concentration ratio of *N*_*Fe*_:*N*_*Pd*_ ≈ 6:1). Each sample vessel was then periodically vortexed for ≈1 second over a 3-hour period, allowed to rest for 48-hours, and then continuously circulated through the magnetic flow cell system for ≈30 minutes at a flowrate of 7–14 mL/min (40–80 volume exchanges). After circulating through the device, the sample was returned to vessel and another 1 mL sub-sample was collected via pipette and transferred to a 15 mL polypropylene tube. The 1 mL sub-samples collected before the addition of the HDTMS-FeNPs and after circulating through the magnetic flow cell were diluted to ≈5 mL using 1% v/v ultrapure (UP) H NO_3_ (Fischer Chemical; Optima Grade, > 99.5%) for plastic digestion and subsequent metals analysis. After processing a given sample, the 10 kDa RC membrane was removed from the magnetic flow cell, dried overnight in a vacuum desiccator and transferred to a Teflon digestion vessel and digested in 10 mL 1:1 UP HNO_3_:DDI at 180 °C for 30 minutes using a MARS 5 Microwave System (CEM). After digestion, the digestate was transferred to a 50 mL polypropylene tube and then diluted to ≈50 mL via triple-rinsing the digestion vessel with DDI and transferring the rinsate to the sample tube. To achieve an appropriate metal concentration for subsequent metals analysis, a 3 mL sub-sample of the diluted digestate was then transferred to a 15 mL polypropylene tube and then diluted to ≈15 mL with DDI. The initial and final sample volumes in each vessel, as well as the volume of the sub-samples before and after dilution, were determined gravimetrically to perform an accurate mass balance of the experimental system and determine the extraction efficiency of the tests (see “performance metrics”, Fig. 1).

Each of the diluted sub-samples were analyzed by inductively coupled plasma mass spectrometry (ICP-MS) (NexIon 350-D; Perkin Elmer) to measure the Pd concentration as a proxy for the nanoplastic concentration. The sub-samples collected before the addition of the HDTMS-FeNPs and after circulating through the magnetic flow cell were compared to measure the decrease in concentration of the PAN-Pd@PS NPs in the samples after introducing the HDTMS-FeNPs and processing the samples with the magnetic flow cell (Fig. 1). In addition, the mass of PAN-Pd@PS NPs extracted on the RC membrane was calculated and compared to the initial mass of PAN-Pd@PS NPs added to the initial sample vessel to quantify the extraction efficiency (Fig. 1). Finally, duplicate samples of each media were prepared and analyzed via the same experimental methods, except that the HDTMS-FeNPs and NaCl were not added to the sample media. These.

## Results and discussion

### Particle characterization

**Fig. 2:**
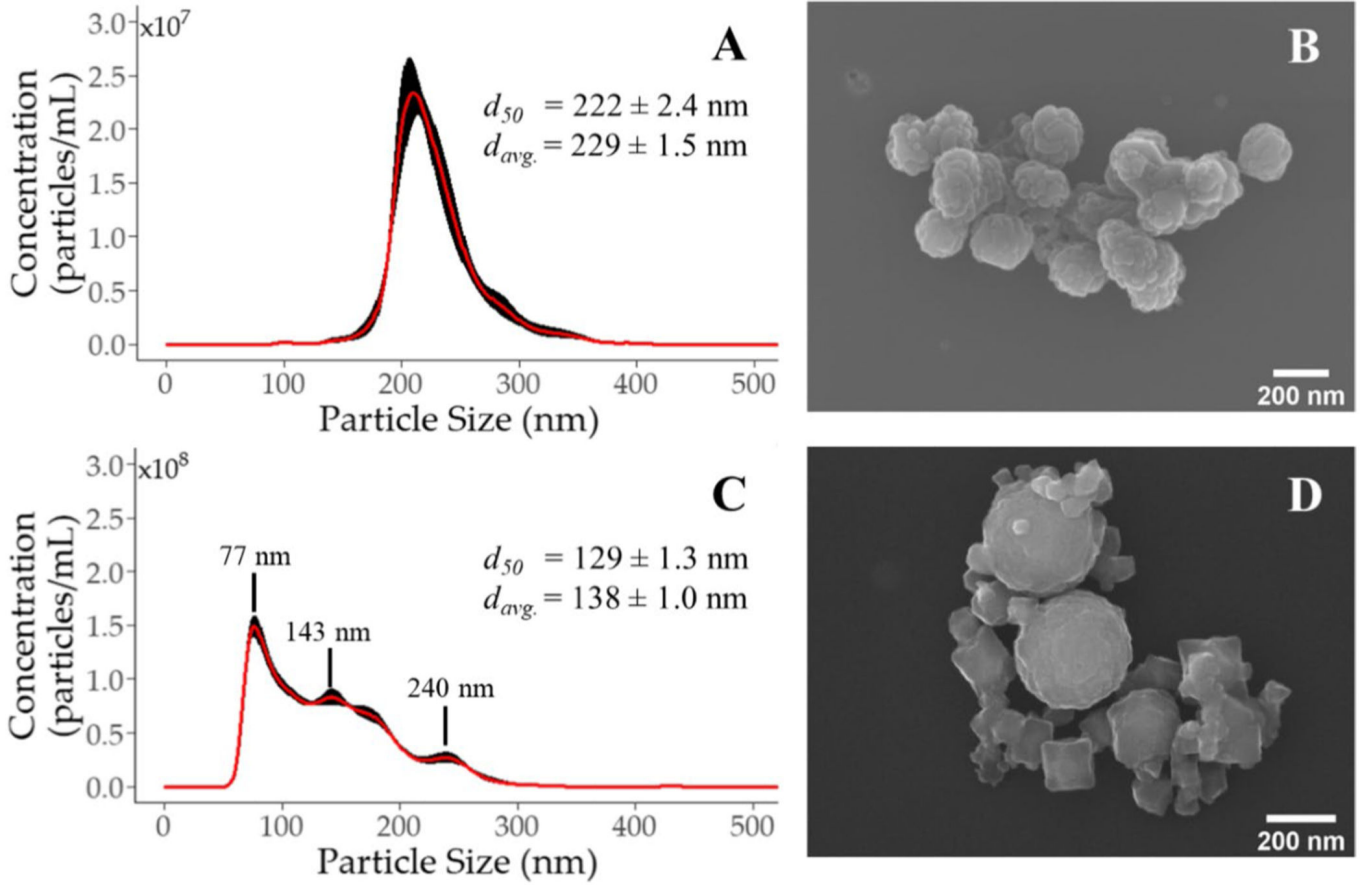
Particle size distribution (PSD) and SEM micrograph of (**a**, **b**) PAN-Pd@PS NPs and (**c**, **d**) HDTMS-FeNPs, respectively. Error bars (black) indicate ±1σ (*n* = 5)

The particle size distribution (PSD) of the PAN-Pd@PS NPs measured via NTA showed a narrow size distribution with an average particle diameter (*d*_*avg*_) of 229 ± 1.5 nm (± 1σ; Fig. 2a). These findings are in-line with both the *Z*-average hydrodynamic diameter (*d*_*h*_) and polydispersity measured via DLS (Table 1), revealing that the PAN-Pd@PS NPs are monodisperse. The electrophoretic mobility (EPM) measured via PALS indicate the PAN-Pd@PS NPs have a negative surface charge (Table 1). The SEM micrographs (Fig. 2b) show a rough surface morphology.

The PSD of the HDTMS-FeNPs had a wider size distribution compared to the PAN-Pd@PS NPs, with *d*_*avg*_ = 138 ± 1.0 nm and the presence of three main size peaks (Fig. 2c). These peaks likely correspond with the size of primary aggregates (ca. 75 nm) and secondary aggregates (ca. 140 and 240 nm) that form during HDTMS-FeNPs synthesis (Table 1). The polydispersity measured via DLS shows that the HDTMS-FeNPs are polydisperse (Table 1), consistent with broader size distribution observed via NTA. The SEM micrographs show a diverse particle morphology, ranging from spherical to cubic-like structures, and a broad range in particle sizes (Fig. 2d and Fig. S2). The EPM measured via PALS indicates that the HDTMS-FeNPs have a negative surface charge (Table 1).

### Method development and optimization

**Fig. 3:**
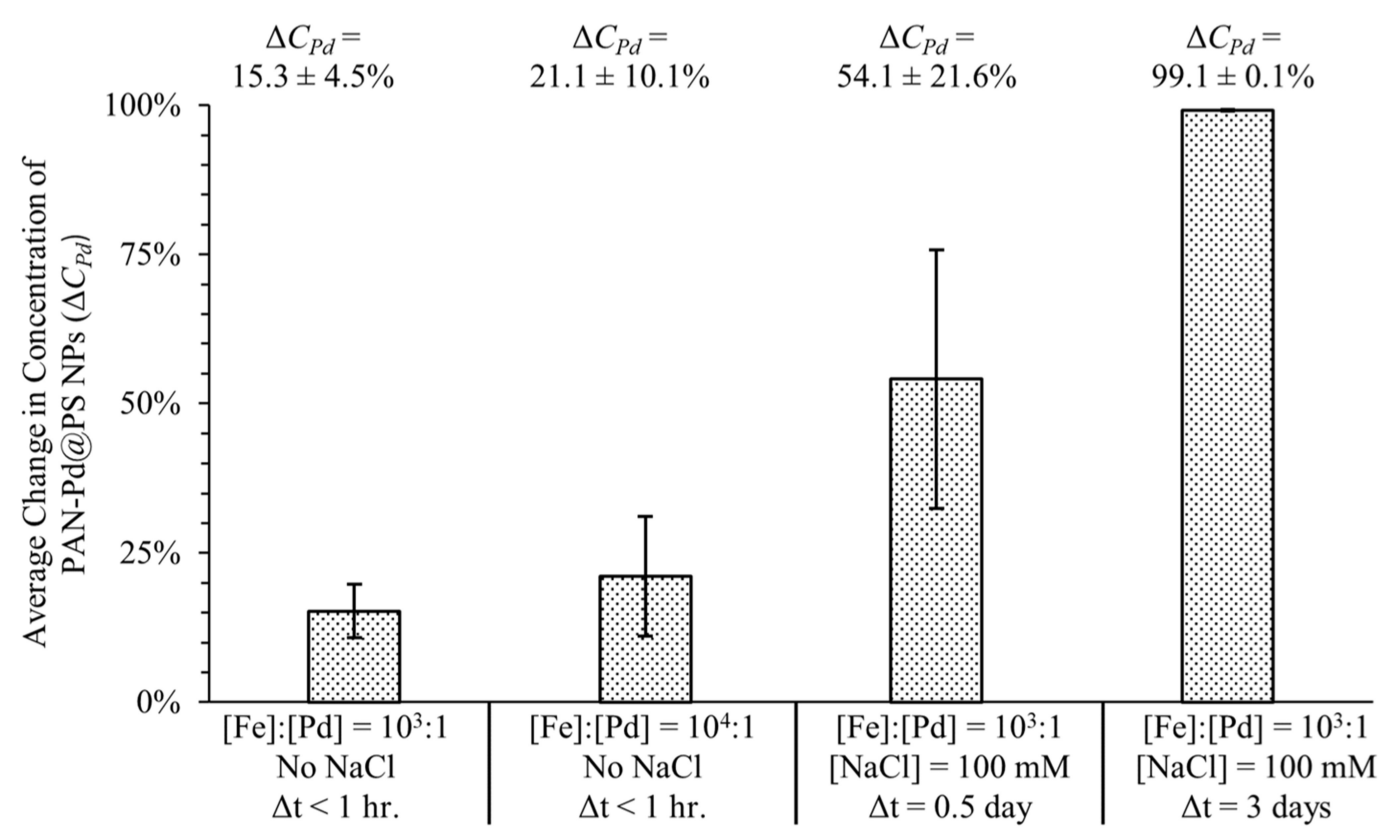
Conditions for optimal removal of PAN-Pd@PS NPs. Average decrease in concentration of PAN-Pd@PS NPs in suspension (Δ*C*_*Pd*_) in response to varying [Fe]:[Pd] mass concentration ratio, concentration of NaCl, and interaction time between HDTMS-FeNPs and PAN-Pd@PS NPs. Error bars indicate ±1σ (*n* = 6)

At a ratio of [Fe]:[Pd] = 10^3^:1 and in the absence of NaCl, resulted in a small decrease in the average concentration of PAN-Pd@PS NPs in suspension was observed after Δ*t* = 1 hour of 15.3 ± 4.5% (Fig. 3). Increasing [Fe]:[Pd] from 10^3^:1 to 10^4^:1 (i.e., increasing *N_Fe_*:*N_Pd_* from ≈3:1 to ≈30:1), again without any NaCl, increased the removal ≈30:1), again without any NaCl, increased the removal of PAN-Pd@PS NPs after Δ*t* = 1 hour to 21.1 ± 10.1% (Fig. 3). This trend is consistent with the second-order dependence of aggregation rates on particle number concentration [28], and is indicative of the HDTMS-FeNPs’ hydrophobicity. A larger decrease in the PAN-Pd@PS NP concentration was observed with the addition of NaCl and when the nanoplastic and iron particles interacted for a longer time period (Fig. 3). Maintaining [Fe]:[Pd] at 10 ^3^:1 but increasing the ionic strength to *I* = 100 mM and allowing the particles to interact for either Δ*t* = 12 or 72 hours increased the change in PAN-Pd@PS NP concentration to 54.1 ± 21.6% and 99.1 ± 0.1%, respectively (Fig. 3).

Initially, it was hypothesized that interactions between HDTMS-FeNPs and PAN-Pd@PS NPs would be primarily driven by hydrophobic forces alone, based on the results reported by Grbic et al. (2019) [22]. However, the higher removal of the PAN-Pd@PS NPs observed following the addition of NaCl (Fig. 3) reveals that other forces, beyond hydrophobicity, influence interactions between the HDTMS-FeNPs and the nanoplastics. To examine this further, a suite of samples were prepared at varying concentrations of NaCl. Between *I* = 20–120 mM, the HDTMS-FeNPs and PAN-Pd@PS NPs were consistently found to heteroaggregate after ≈1–2 hours (Fig. S3a–f). Given the negative surface charge of the HDTMS-FeNPs and PAN-Pd@PS NPs, as well as the trends in the PAN-Pd@PS NP removal discussed above, the behavior of the particles in response to increasing concentrations of NaCl is indicative of charge screening effects likely due to electric double layer (EDL) compression [29–31]. Time resolved dynamic light scattering (TR-DLS) measurements performed over a 24-hour period at *I* = 90 mM showed that the PAN-Pd@PS NPs homoaggregate slowly, with an aggregation rate (d [*d*_*h*_]/d [*t*]) between 4.9–6.9 nm/minute (detailed in Supplementary Information and Fig. S4). In combination, these results demonstrate that the interaction of the HDTMS-FeNPs with the nanoplastic particles used in our experimental system is dependent on both the hydrophobicity of the HDTMS-FeNPs as well as charge screening effects (i.e., increased compression of the EDL with increasing ionic strength).

### Nanoplastics extraction tests using a magnetic flow cell

**Fig. 4:**
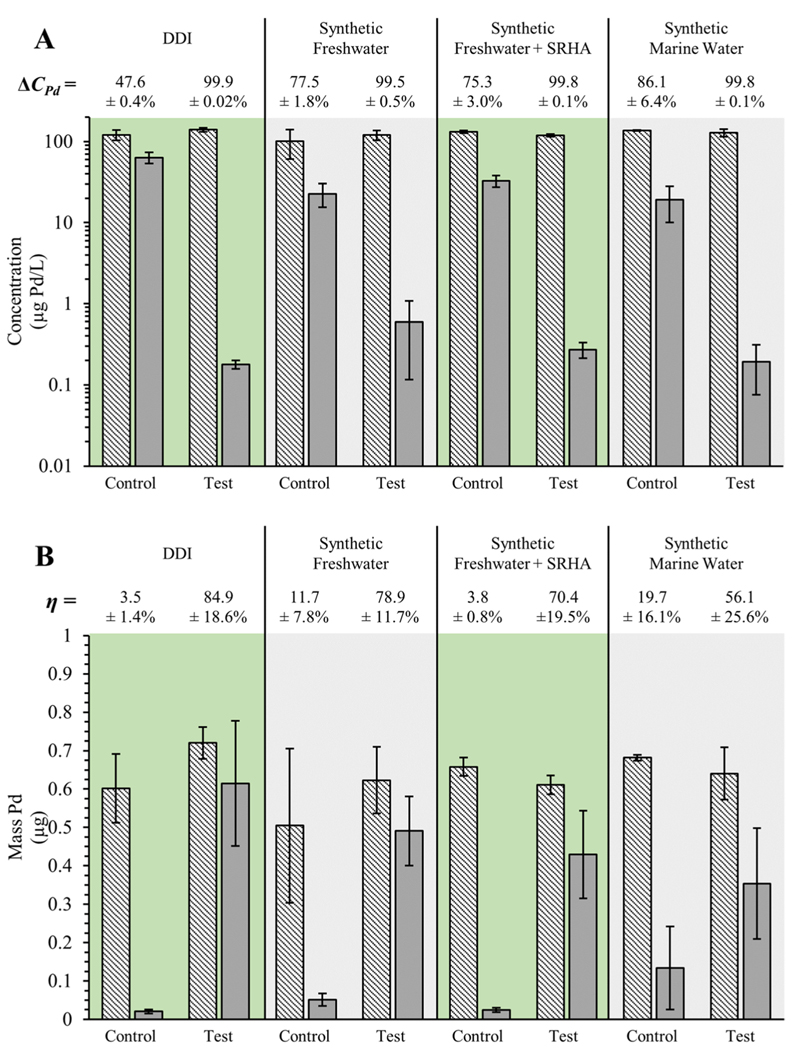
PAN-Pd@PS NPs removed from suspension (**A**) and captured on the membrane (**B**) using the Magnetic Flow Cell. **A** Average initial concentrations (hashed) and final concentrations (solid) of PAN-Pd@PS NPs in each aquatic media after Δ*t* = 48 hours and (**B**) average mass of PAN-Pd@PS NPs in the initial sample (hashed) and extracted onto 10 kDa RC membrane (solid). “Control” indicates samples without NaCl and HDTMS-FeNPs while “test” indicates samples with NaCl and/or HDTMS-FeNPs. Error bars indicate ±1σ (Test: *n* = 6; Control: *n* − = 2)

Using the results of the optimization tests, the first set of extraction tests using the magnetic flow cell were conducted in DDI. This was done to help identify any artifacts introduced by the extraction system and to establish the baseline performance of the system under idealized conditions. Across the six replicates prepared in DDI, a decrease in the concentration of PAN-Pd@PS NPs in suspension was consistently observed after Δ*t* = 48 hours (Fig. 4a), with an average decrease of 99.9 ± 0.02% (±1σ, *n* = 6). Comparing the average mass of the PAN-Pd@PS NPs in the initial sample vessels with that extracted on the 10 kDa RC membrane indicates a high extraction efficiency of 84.9 ± 18.6% (±1σ, *n* = 6; Fig. 4b). Images demonstrating the particle aggregates formed via this technique and subsequently extracted with the magnetic flow cell are provided in the Supplementary Information (Fig. S3g). In combination, these results indicate that the addition of the HDTMS-FeNPs and NaCl followed by circulation of the dispersion through the magnetic flow cell can effectively extract and concentrate the nanoplastics onto the membrane.

The effectiveness of the extraction technique was further evaluated in a suite of synthetic aquatic media intended to mimic increasingly realistic environmental aqueous media. In synthetic freshwater without NOM, which contained both mono- and divalent ions at environmentally relevant concentrations (see Supplementary Information Table S1), the decrease in PAN-Pd@PS NPs concentrations was 99.5 ± 0.5% while the extraction efficiency was 78.9 ± 11.7% (±1σ, *n* = 6; Fig. 4a and b, respectively). With the addition of the model NOM (SRHA), a comparable decrease in PAN-Pd@PS NPs concentrations was observed (99.8 ± 0.1%) while the extraction efficiency was reduced to 70.4 ± 19.5% (±1σ, *n* = 6; Fig. 4a and b, respectively). Finally, in synthetic marine water, which did not contain NOM but had substantially higher concentrations of both mono- and divalent ions compared to synthetic freshwater, PAN-Pd@PS NPs concentration was similar to the results observed in the synthetic freshwaters (99.8 ± 0.1%) while the extraction efficiency further decreased to 56.1 ± 25.% (±1σ, n = 6; Fig. 4a and b, respectively). Differences between PAN-Pd@PS NPs removed from suspension and captured onto the 10 kDa RC membrane appear to be bound in the system (i.e., tubing and flow cell).

The suitability of the magnetic flow cell system to effectively extract and concentrate nanoplastics was found to be dependent on the chemical composition of the sample media. The results shown in Fig. 4a demonstrate that the PAN-Pd@PS NPs are consistently lost from suspension across all four media examined. However, the decreasing extraction efficiency when progressing from idealized (DDI) to more environmentally relevant conditions (Fig. 4b) indicates that the additional constituents in the environmentally representative media affects heteroaggregation of the HDTMS-FeNPs and PAN-Pd@PS NPs and thus their extractability within the magnetic flow cell. For example, comparing the extraction efficiency in the synthetic freshwater with and without NOM, the decreased extraction performance in the presence of NOM is likely due to the adsorption of NOM macromolecules onto the surface of the HDTMS-FeNPs and/or PAN-Pd@PS NPs. This is a well-established phenomenon that is known to affect and even prevent particle aggregation [32–36]. In the synthetic marine water, the reduced extraction efficiency is likely due to the high ionic strength of the media. Based on visual observations and long duration TR-DLS measurements, the HDTMS-FeNPs and PAN-Pd@PS NPs were found to heteroaggregate at an ionic strength as low as 20 mM (see Supplementary Information Fig. S3). At the ionic strength of the synthetic marine water (≈665 mM; Table S1), the PAN-Pd@PS NPs would be expected to undergo rapid homoaggregation and may have settled out of suspension prior to the addition of the HDTMS-FeNPs. Without nanoplastic heteroaggregation with HDTMS-FeNPs, these nanoplastics would not be removed within the magnetic flow cell.

It may also be possible that some portion of the HDTMS-FeNP and PAN-Pd@PS NP heteroaggregates were unintentionally removed by the components of the magnetic flow cell itself, a potential artifact of the systems’ design. For example, in duplicate control samples prepared in DDI but without the addition of the HDTMS-FeNPs or NaCl, the PAN-Pd@PS NPs concentration decreased 47.6 ± 0.4% while the extraction efficiency was only 3.5 ± 1.4% (±1σ, *n* = 2; Fig. 4a and b, respectively). This indicates that in the absence of the HDTMS-FeNPs and NaCl, a portion of the PAN-Pd@PS NPs are lost to the vessel walls and/or tubing, with a negligible amount unintentionally adsorbed onto the membrane (Fig. 4a and b). Similar trends were observed in the environmentally relevant media. In relation to the control tests performed in DDI, higher removal of the PAN-Pd@PS NPs was observed in the synthetic freshwater media in both the presence and absence of NOM (77.5 ± 1.8% and 75.3 ± 3.0%, respectively; ±1σ, *n* = 2; Fig. 4a), with the highest seen in the synthetic marine water (86.1 ± 6.4%; ±1σ, *n* = 2; Fig. 4a). This trend reflects the effect of the ionic species inherent to the synthetic environmental media and their effect on the colloidal stability of the PAN-Pd@PS NPs. However, in each of the environmentally relevant media tested, relatively small extraction efficiencies were observed, with 11.7 ± 7.8%, 3.8 ± 0.8%, and 19.7 ± 16.1% (±1σ, *n* = 2) in the synthetic freshwater, synthetic freshwater with NOM, and synthetic marine water, respectively (Fig. 4b). The decrease in extraction efficiency observed in the synthetic freshwater in the presence of NOM is attributed to the stabilizing effect of NOM adsorption on the PAN-Pd@PS NPs, minimizing their adsorption to the vessel walls and/or tubing as well as the membrane. Thus, the trends in PAN-Pd@PS NPs concentration and extraction efficiency found with the control samples demonstrate the tendency for the PAN-Pd@PS NPs to adsorb with the sample vessel walls and/or tubing in the absence of the HDTMS-FeNPs and added NaCl.

Nevertheless, the substantially higher extraction efficiency observed following the addition of the HDTMS-FeNPs (and NaCl, when added) relative to that observed in the control tests clearly demonstrates the effect that HDTMS-FeNPs have on the extraction of the nanoplastics onto the membrane. The extraction efficiencies reported in Fig. 4b indicate that the combination of the magnetic nanoparticles and flow cell system is a promising technique to extract and concentrate nanoplastic samples from aquatic media. Based on the trends in the extraction efficiency, this method may be better suited to relatively “clean” samples, such as drinking water or low turbidity surface water samples, as opposed to more complex samples containing more background particles or those with higher ionic strengths, such highly turbid surface waters or marine water.

### Implications and future research

From a broad perspective, our results demonstrate that the behavior of plastic particles across the nano- to microplastic size scale may differ in response to similar physical phenomena. For example, the results presented by Grbic et al. (2019) [22] demonstrated that the hydrophobically-functionalized HDTMS-FeNPs effectively extracted small-to-large microplastic particles across a diverse range of media with varying polymer chemistries. In this size range, the hydrophobicity of the HDTMS-FeNPs was sufficient to drive their adsorption with microplastic particles, thus enabling their extraction via an applied magnetic field. In contrast, our results suggest that for the nanoplastics we tested, the hydrophobicity of the HDTMS-FeNPs was unable to cause attachment to the model nanoplastics used in our research, highlighting the importance of also considering electrostatic forces that become increasingly important at smaller particle sizes and considering that plastic particles of varying polymer chemistry may exhibit differing surface charges. Another difference between the magnetic separation behavior for micro-and nanosized plastic particles may result from the number of HDTMS-FeNPs which would be required to bind to and occupy the surface of larger (micro) versus smaller (nano) plastic particles. Modeling results suggest that the maximum relative number of HTTMS-FeNPs that could occupy the surface of a PAN-Pd@PS NP would be approximately 1 0^3^ times lower than the number that could occupy the surface of a 10 μm plastic particle (see Supplementary Information and Fig. S5). Thus, the magnetic force “pulling” a nanoplastic particle from suspension using our technique would be less than that applied to a much larger microplastic particle.

While our research was limited to a single nanoplastic polymer type (PS), the findings may translate to other environmentally relevant polymer types (e.g., polyethylene, polypropylene, polyethylene terephtalate, etc.). However, further testing would be needed to evaluate the effectiveness of our extraction method on other polymer types due to differences in their inherent hydrophobicity and surface charge relative to the model nanoplastics we used. As advancements in nanoplastic analytical methods develop further, thus enabling their detection without the need to rely on embedded metallic tracers, it would be worthwhile to repeat these tests to understand the broader applicability of this method to other nanoplastic polymer types. In regard to the aquatic chemistry of the suspending medium, our work demonstrates that constituents likely to be encountered in environmental samples (i.e., mono- and divalent ions, NOM) can impact the effectiveness of this method. Thus, future research should look to further examine both the impact of these constituents on this technique, as well as modifications to the approach that may mitigate their impacts. For example, adjustments to the mixing duration or amount of HDTMS-FeNPs added to (and thus the ratio of *N*_*Fe*_:*N*_*NP*_) to the samples could be examined to ascertain whether these factors can improve the extraction efficiency of this method. It is also plausible that media-specific operating parameters may be elucidated (e.g., reducing the interaction time for marine water samples and increasing the duration for freshwater samples). In general, it is anticipated that further testing and refinement of the operating parameters would be needed, especially if the method were to be scaled-up with the intention of field-testing this method. For example, the current amount of HDTMS-FeNPs added to the samples would be prohibitive if the sample volume were increased beyond the relatively small volumes currently tested.

Given the challenges with separating and concentrating nanoplastic particles from the environment, this method represents a proof of principle that could be applied after removing chemically digestible biomaterial and dense geoparticles. Finally, this research was completed using idealized conditions, including only two particle types within the experimental system (HDTMS-FeNPs and PAN-Pd@PS NPs). It is intended to be applied to separate and concentrate nanoplastics from environmental samples but only in the context of a more comprehensive pre-treatment regime. For example, it is unclear what potential impact the presence of non-target bio- and geogenic natural colloids, which are ubiquitous in the environment, may have on the effectiveness of this technique. This should be examined, potentially through the use of model natural colloids such as hematite or bentonite; however, as this technique is intended to be a component within a broader sample preparation scheme, it is expected that some of these interferences would be reduced or eliminated. For example, a combination of clean-up techniques, such as tangential flow filtration, density gradient centrifugation, and/or digestion methods (e.g., enzymatic) could be used to reduce the amount of non-target constituents in the sample. However, care would need to be taken to ensure that these methods do not inadvertently remove the target nanoplastics. Although, some of these methods may be limited to laboratory operation, continuous centrifugation [37] has been shown to separate and concentrate plastic nanoparticles in environmental media at flow rates of up to 5 L min^−1^. For a field deployable system, it is envisioned that combining a flow-through filtration technique (e.g., tangential flow filtration) with the magnetic extraction technique described here could be highly effective when field sampling nanoplastics. In such a set-up, a series of tangential flow filters could be used to separate particles ≤1 μm from particles > 1 μm, with each fraction processed using a magnetic flow cell system tailored to isolate and extract plastic particles from the different size ranges. By increasing the relative concentration of nanoplastics in a smaller volume, the addition of HTTMS-Fe NPs at the concentrations that we report would be more practical as compared to the large volumes typical of environmental samples.

The research reported here indicate that magnetic nanoparticles in combination with a flow-through system is a promising technique to extract nanoplastics from aqueous suspensions with various compositions. Through further refinement and testing, such as using more complex sample matrices, a lower concentration of nanoplastic particles, and more environmentally representative nanoplastics, it is envisioned that the utility of this method can more fully evaluated. In combination with other sample reduction or clean-up techniques, insights regarding the nature and extent of nanoplastic pollution in the environment may be possible.

## Supplementary Material

Supplement1 **Figure S1**. (top) Illustration depicting material flow and operation of magnetic flow cell system and a photograph of (bottom) the major components of the magnetic flow cell.
**Figure S2**. Energy dispersive X-ray (EDX) analysis of HDTMS-FeNPs.
**Figure S3**. Heteroaggregates of HDTMS-FeNPs and PAN-Pd@PS NPs (a-f) formed after Δ*t* = 1–2 hours in varying concentrations of NaCl and (g) aggregates subsequently extracted onto 10 kDa regenerated cellulose membrane following circulation of dispersion through the magnetic flow cell (photo depicts subset of samples).
**Figure S4**. Duplicate TR-DLS measurements demonstrating the change in Z-average hydrodynamic diameter (*d*_*z-avg*_) with time of PAN-Pd@PS NP_s_ dispersed in / ≈ 90 mM NaCl. Linear regression fit to data between t = 0 – ≈500 minutes.
**Figure S5**. Theoretical number of HDTMS-FeNPs adsorbed to spherical plastic particles of varying size based upon Random Sequential Adsorption model. The *d*_*avg*_ of the PAN-Pd@PS NPs measured via NTA (229 ± 1.3 nm; Table 1) is indicated.
**Table S1**. Summary of synthetic media water quality.

## Figures and Tables

**Table 1:** Characteristics of PAN-Pd@PS NPs and HDTMS-FeNPs

Parameter	PAN‑Pd@PS NPs	HDTMS‑FeNPs
Z-Average Hydrodynamic Diameter (*d_h_*) (nm)	258.1 ± 4.3	178.6 ± 1.9
Polydispersity Index (PDI)	0.07 ± 0.03	0.09 ± 0.06
Electrophoretic Mobility (EPM) ([μm/s]/[V/cm])	−3.03 ± 0.22S	−1.61 ± 0.46
Zeta Potential (ζ)^a^ (mV)	−38.61 ± 2.79	−20.59 ± 5.82

Error bars indicate ±1σ (*d_h_*: *n* = 6; EPM/ζ: *n* = 10).

a Reported by instrument using Hückel Model; value not adjusted for pH/ionic strength of dispersion
