# Supplementary material for: Extraction and concentration of nanoplastic particles from aqueous suspensions using functionalized magnetic nanoparticles and a magnetic flow cell
Source: Microplast nanoplast. Author manuscript; Available in PMC 2024 Jan 27. (PMC10624164; doi:10.1186/s43591-022-00051-1)
Supplement: Supplement1 — Figure S1. (top) Illustration depicting material flow and operation of magnetic flow cell system and a photograph of (bottom) the major components of the magnetic flow cell. Figure S2. Energy dispersive X-ray (EDX) analysis of HDTMS-FeNPs. Figure S3. Heteroaggregates of HDTMS-FeNPs and PAN-Pd@PS NPs (a-f) formed after Δt = 1–2 hours in varying concentrations of NaCl and (g) aggregates subsequently extracted onto 10 kDa regenerated cellulose membrane following circulation of dispersion through the magnetic flow cell (photo depicts subset of samples). Figure S4. Duplicate TR-DLS measurements demonstrating the change in Z-average hydrodynamic diameter (dz-avg) with time of PAN-Pd@PS NPs dispersed in / ≈ 90 mM NaCl. Linear regression fit to data between t = 0 – ≈500 minutes. Figure S5. Theoretical number of HDTMS-FeNPs adsorbed to spherical plastic particles of varying size based upon Random Sequential Adsorption model. The davg of the PAN-Pd@PS NPs measured via NTA (229 ± 1.3 nm; Table 1) is indicated. Table S1. Summary of synthetic media water quality. [file NIHMS1919173-supplement-Supplement1.docx]

Supplementary Information

Extraction and Concentration of Nanoplastic Particles from Aqueous Suspensions using Functionalized Magnetic Nanoparticles and a Magnetic Flow Cell

Mark C. Surette^1,2^, Denise M. Mitrano^3^, and Kim R. Rogers^4*^

*Corresponding Author: [Rogers.Kim@epa.gov](mailto:Rogers.Kim@epa.gov)

^1^ ORISE Postdoctoral Research Participant at

U.S. EPA Center for Environmental Measurement and Modeling

109 T.W. Alexander Drive, Research Triangle Park, NC 27709

^2^ Current Address: WSP USA Solutions, Inc.,

18300 NE Union Hill Road Suite 200, Redmond, WA 98052

^3^ Department of Environmental Systems Science

Institute of Biogeochemistry and Pollutant Dynamics

ETH Zürich, 8092 Zürich, Switzerland

^4^ U.S. EPA Center for Environmental Measurement and Modeling

109 T.W. Alexander Drive, Research Triangle Park, NC 27709

**8** Pages

**1** Tables

**5** Figures

Magnetic Flow Cell Components / Operation and Experimental Protocol

A graphic illustrating the fluid flow and a close-up of the interior of the flow channel is provided in Figure S1 (top), with the major components of the magnetic flow cell shown in Figure S1 (bottom). The 10 kDa regenerated cellulose membrane was placed underneath the channel spacer and directly on top of the bottom portion of the enclosure shown in Figure S1. In this position, the dispersion only flows over the top of the membrane before exiting the channel (i.e., there is no flow through the membrane), with the membrane acting as a removable surface that particles are extracted onto.


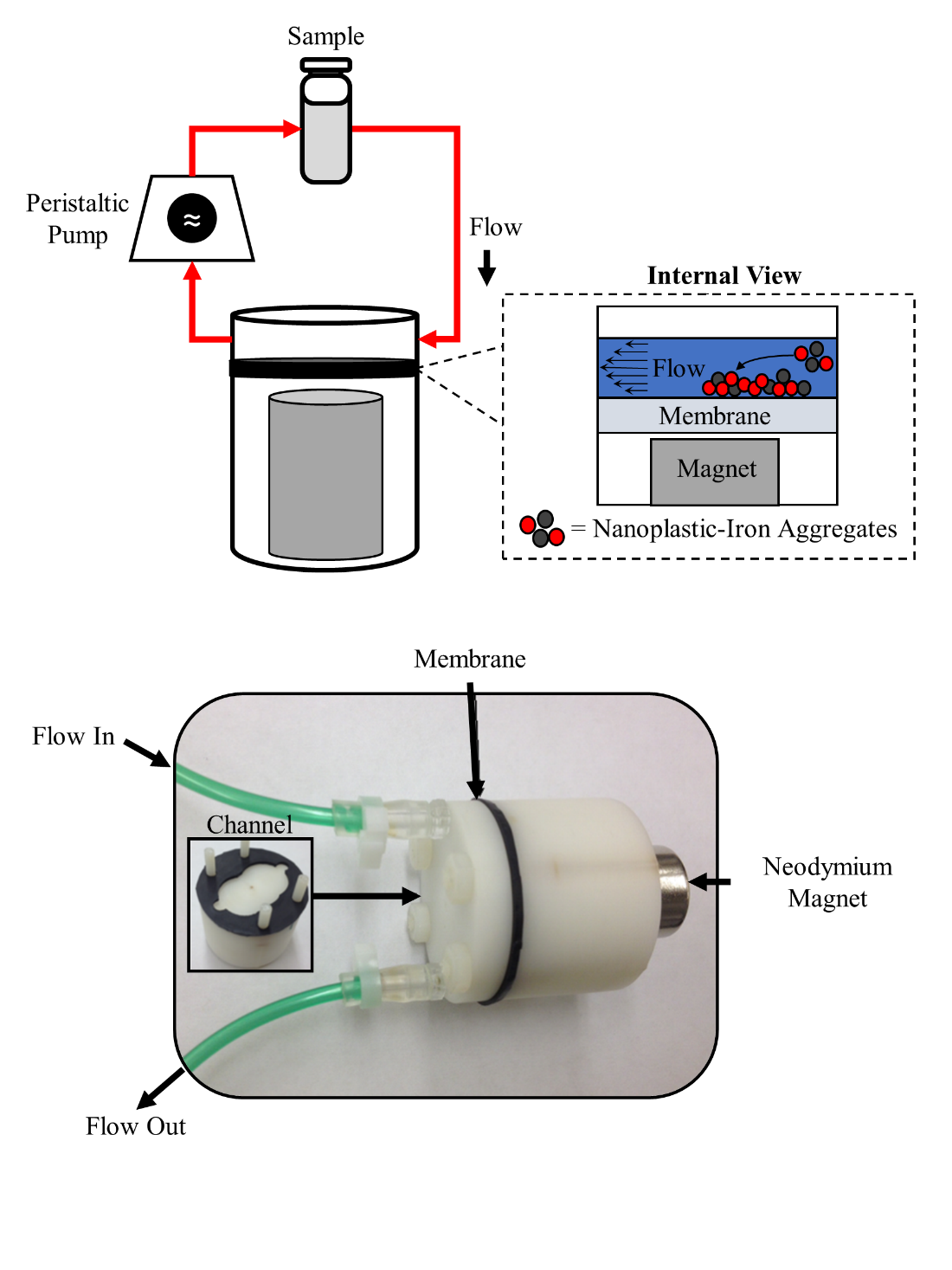


**Figure S1.** (top) Illustration depicting material flow and operation of magnetic flow cell system and a photograph of (bottom) the major components of the magnetic flow cell.

Preparation and Constituents of Synthetic Aquatic Media

Per the methods detailed in *Methods for Measuring the Acute Toxicity of Effluents and Receiving Waters to Freshwater and Marine Organisms 5th Edition* (U.S. EPA, 2002)^1^, the synthetic freshwater was prepared according to the protocol for Standard, Synthetic Freshwater (Section 7.2.3), with ionic species at the concentrations specified under moderately hard water (MHW), while the synthetic marine water (InstantOcean®) was prepared according to the protocol for Standard, Synthetic Seawater (Section 7.2.4), with the amount of InstantOcean® added equal to a salinity of 31 parts per thousand (ppth). The Suwannee River Humic Acid (SRHA) was prepared according to the manufacture’s recommended protocol to a total organic carbon (TOC) concentration ≈50 mg C/L and then diluted in the synthetic freshwater media to obtain a [TOC] = 1 mg C/L. A summary of the general water quality characteristics of the synthetic freshwater and marine water are provided in Table S1.

**Table S1.** Summary of synthetic media water quality.

| **Parameter** | **Synthetic Freshwater** | **Synthetic Freshwater**  **+ SRHA** | **Synthetic Marine Water** |  |
| --- | --- | --- | --- | --- |
| pH | 7.4 – 7.8 | 7.4 – 7.8 | 8.4 |  |
| Hardness | 80 – 100 | 80 – 100 |  | mg CaCO_3_/L |
| Alkalinity | 57 – 64 | 57 – 64 |  | mg CaCO_3_/L |
| Total Organic Carbon (TOC) | N/A | 1 | N/A | mg C/L |
| Ionic Strength (*I*) | 3.0 | 3.0 | 665 | mM |
| ***Major Cations*** |  |  |  |  |
| Na+ | 1.15 | 1.15 | 462 | mM |
| K^+^ | 0.05 | 0.05 | 9.4 | mM |
| Mg^2+^ | 0.50 | 0.50 | 53 | mM |
| Ca^2+^ | 0.35 | 0.35 | 9.4 | mM |
| Sr^+^ | N/A | N/A | 0.19 | mM |
| ***Major Anions*** |  |  |  |  |
| Cl^-^ | 0.05 | 0.05 | 521 | mM |
| SO_4_^2-^ | 0.35 | 0.35 | 23 | mM |

Particle Characterization

Given the varied morphology of the HDTMS-FeNPs (see Figure 2d in main text), energy dispersive X-ray (EDX) analysis of the samples was performed concurrently with the collection of the SEM micrographs at an acceleration voltage of 20 kV and a working depth of 5.0 mm. As shown in Figure S2, the Fe-Kα signal is distributed throughout the particle aggregate, indicating that the FeNP morphology is varied (i.e., spherical, plates, and cubic structures) but all particles contain Fe regardless of their morphology.


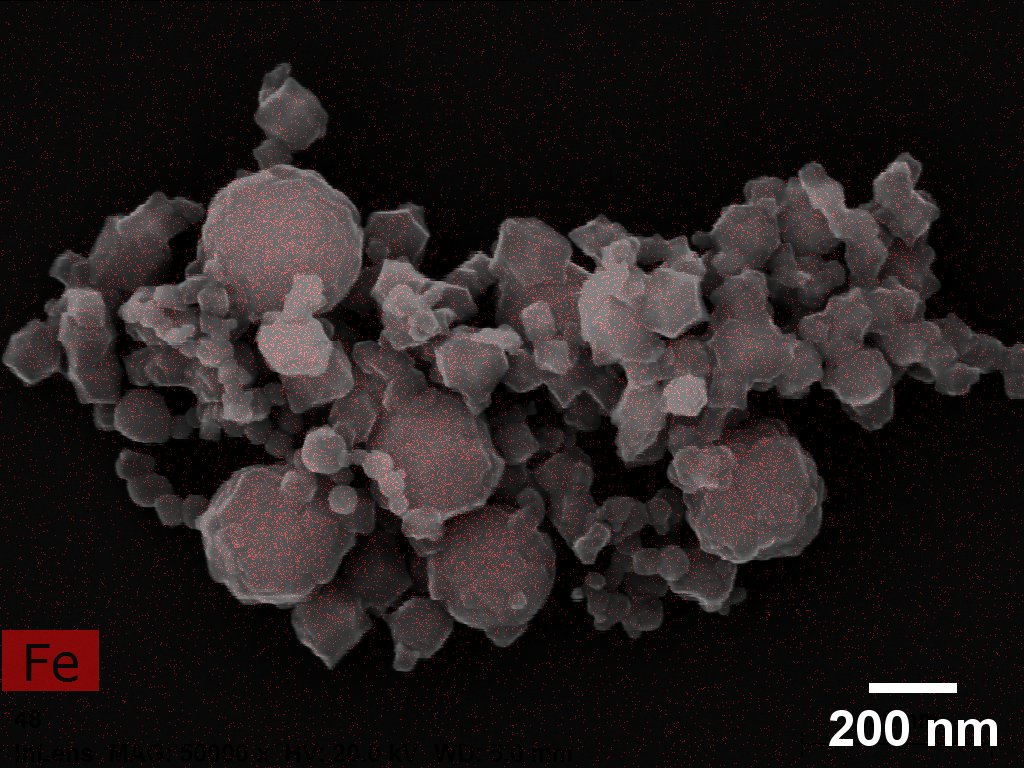

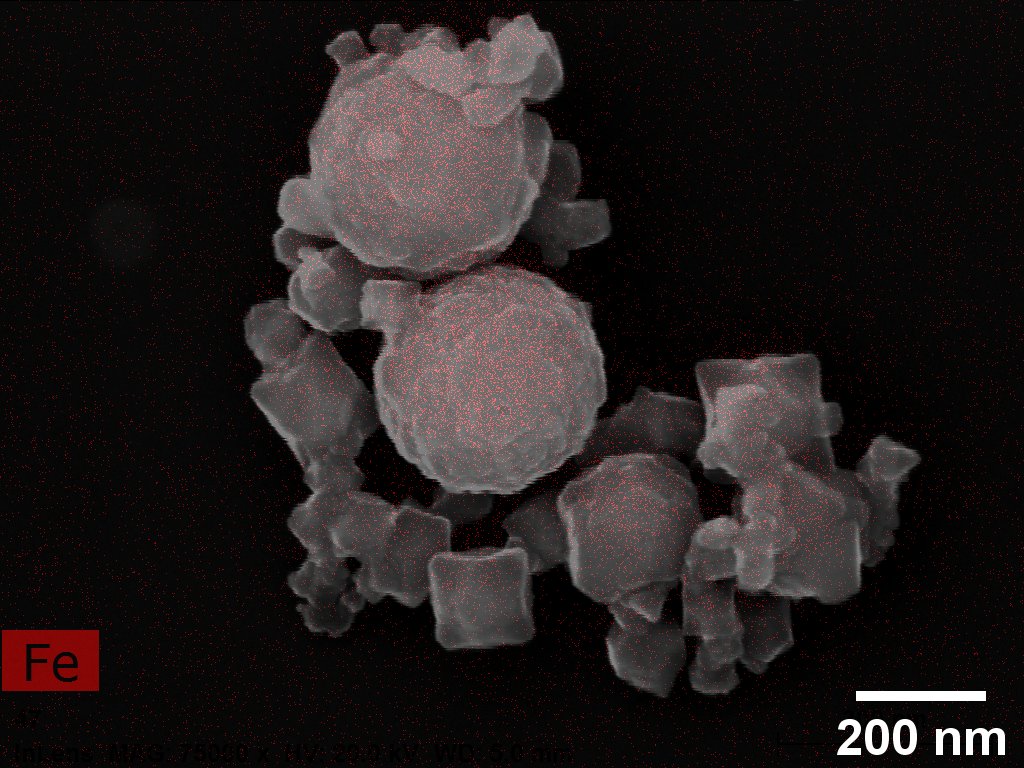


**Figure S2.** Energy dispersive X-ray (EDX) analysis of HDTMS-FeNPs.

Aggregation of HDTMS-FeNPs and PAN-Pd@PS NPs in NaCl

To examine the impacts of varying concentrations of NaCl on the aggregation behavior of the HDTMS-FeNPs and PAN-Pd@PS NPs, a suite of samples were prepared according to initial procedure described in the main text except that the volume of NaCl added was varied to reach *I* = 20 – 120 mM. After adding NaCl, the dispersions were briefly mixed and observed after Δ*t* = 1-2 hours post-NaCl addition. The results, shown in Figure S3a-f clearly demonstrate the presence of heteroaggregates.


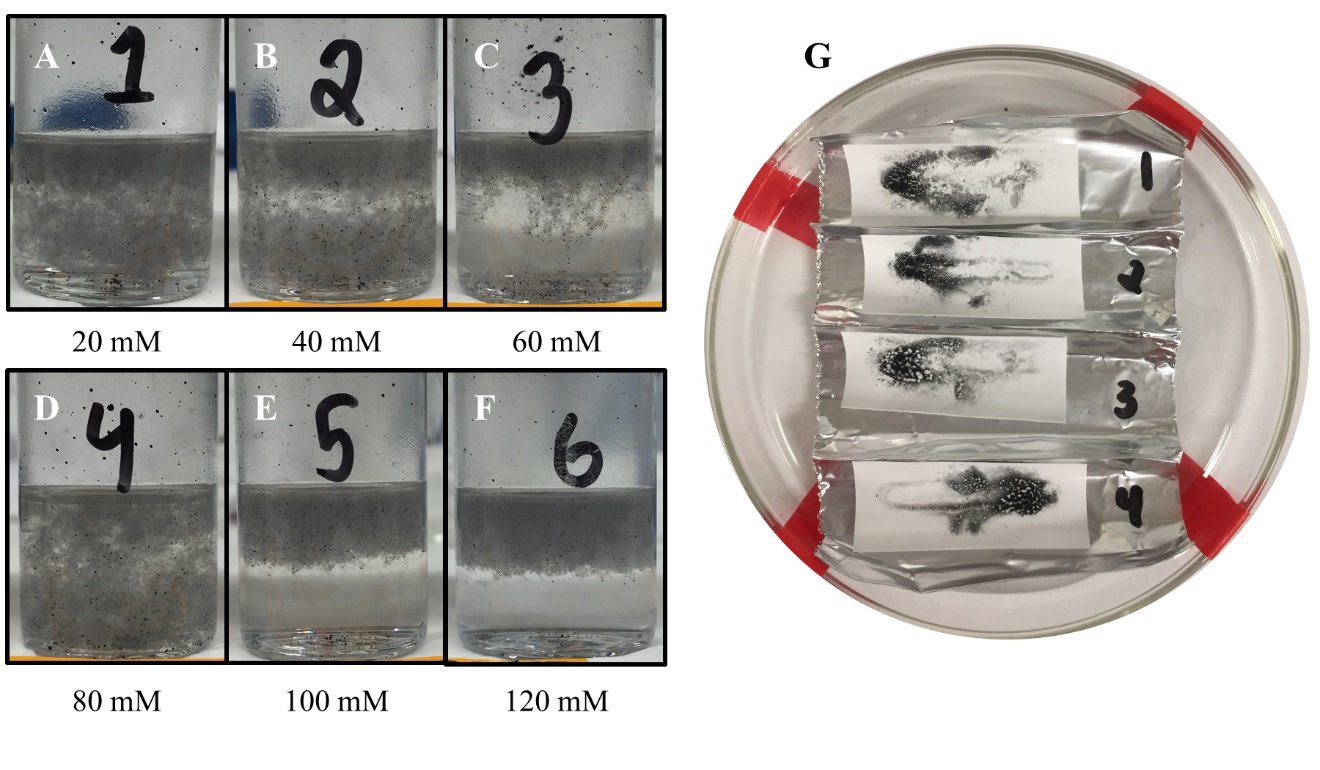


**Figure S3.** Heteroaggregates of HDTMS-FeNPs and PAN-Pd@PS NPs (a-f) formed after Δ*t* = 1-2 hours in varying concentrations of NaCl and (g) aggregates subsequently extracted onto 10 kDa regenerated cellulose membrane following circulation of dispersion through the magnetic flow cell (photo depicts subset of samples).

To further examine the aggregation behavior of the PAN-Pd@PS NPs, longer-duration time resolved dynamic light scattering (TR-DLS) measurements were preformed over a ≈24-hour period. Duplicate samples were prepared (*V_TOT_* = 3 mL) by first combining an aliquot of the PAN-Pd@PS NP dispersion with DDI to reach a nominal concentration of 100 µg Pd/L (mimicking the concentration of PAN-Pd@PS NPs used in the batch tests). Each sample was then analyzed via DLS using a ZetaSizer Nano ZS (Malvern Panalytical) with three measurements per sample (3 runs/measurement at 20 seconds/run) to determine the initial size of the PAN-Pd@PS NPs. An aliquot of 1 M NaCl was then added to the sample (*I* ≈ 90 mM), briefly mixed by inverting the sample, and then inserted into the instrument. The sample was then analyzed over a 24-hour period with 144 measurements per sample (1 run/measurement at 20 sec./run) with a 600-second delay between subsequent measurements. The results indicate that the PAN-Pd@PS NPs homoaggregate over time with increased ionic strength (Figure S4). A linear regression was fit to the data between *t* = 0 – ≈500 minutes to estimate the aggregation rate (d[*d_h_*]/d[*t*]). This period was chosen as increasing variability in the size measurements was observed after *t* ≈500 minutes due to limitations with the analytical technique (i.e., impacts of sedimentation).


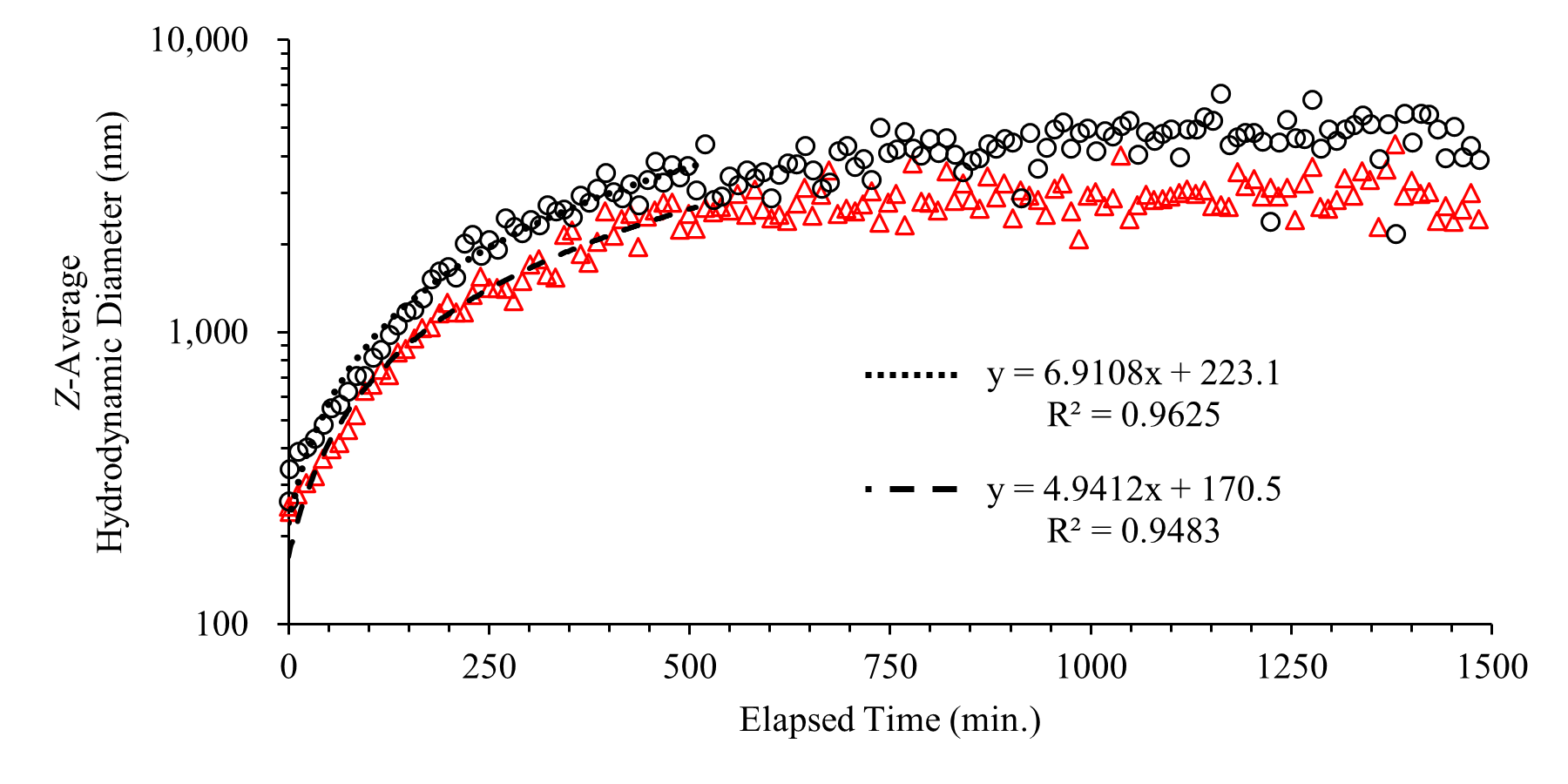


**Figure S4.** Duplicate TR-DLS measurements demonstrating the change in Z-average hydrodynamic diameter (*d_z-avg_*) with time of PAN-Pd@PS NPs dispersed in *I* ≈ 90 mM NaCl. Linear regression fit to data between *t* = 0 – ≈500 minutes.

Modeling the Number of Adsorbed HDTMS-FeNPs to Plastic Particles

The lower extraction efficiency of nanoplastics in the absence of NaCl compared to that observed in tests performed by Grbic et al. (2019)^2^ using microplastic particles may be linked to differences in the size of the particles that were tested. Using the Random Sequential Adsorption (RSA) model detailed by Sadowska et al. (2014)^4^, the number of HDTMS-FeNPs that could theoretically bind to the surface of differently sized plastic particles was estimated. The maximum fractional surface coverage (*θ_max_*) of a spherical plastic particle (of varying sizes) that could be “occupied” by the HDTMS-FeNPs was calculated and used to estimate the maximum number of HDTMS-FeNPs that could bind to the surface. There are a number of assumptions that are required to perform this analysis, including that the model plastic particles are monodisperse spherical collectors and that the HDTMS-FeNPs are also monodisperse with a diameter equal to *d_avg_* measured via NTA (138 ± 1.0 nm; Figure 2 in main text). Although *θ_max_* is calculated by incorporating electrostatic interactions between adjacent HDTMS-FeNPs, it ignores steric interactions that likely occur in practice. The results indicates that a nanoplastic particle equivalent to the size of the PAN-Pd@PS NPs (*d_avg_* = 229 ± 1.3 nm) could ‘accommodate’ ≈10 HDTMS-FeNPs adsorbed on its surface, compared to ≈10^4^ on the surface of a 10 µm microplastic particle (Figure S5).

Relative to micro- and mesoplastic particles, the surface area of each PAN-Pd@PS NP is significantly lower, thus limiting the number of HDTMS-FeNPs that can adsorb to the particle surface due to physical limitations.^3,4^ As the magnetic force exerted on each plastic particle is directly related to the total volume of HDTMS-FeNPs bound to the particle surface (∝ r^3^)^5,6^, the PAN-Pd@PS NPs (as well as other, similar-sized nanoplastic particles) would experience a significantly lower magnetic force relative to micro- and mesoplastic particles. If this magnetic force were less than the hydrodynamic forces within the magnetic flow cell, this would effectively prevent the extraction of nanoplastics onto the membrane within the flow cell (i.e., the hydrodynamic forces of the fluid flow within the flow cell would overcome the magnetic force holding the particles against the membrane). This observation highlights the importance of increasing the ionic strength of the media to promote the heteroaggregation of the PAN-Pd@PS NPs with the HDTMS-FeNPs, thus increasing the total mass of HTDMS-FeNPS bound to each aggregate structure and increasing the effective magnetic force trapping the nanoplastics aggregates onto the membrane.


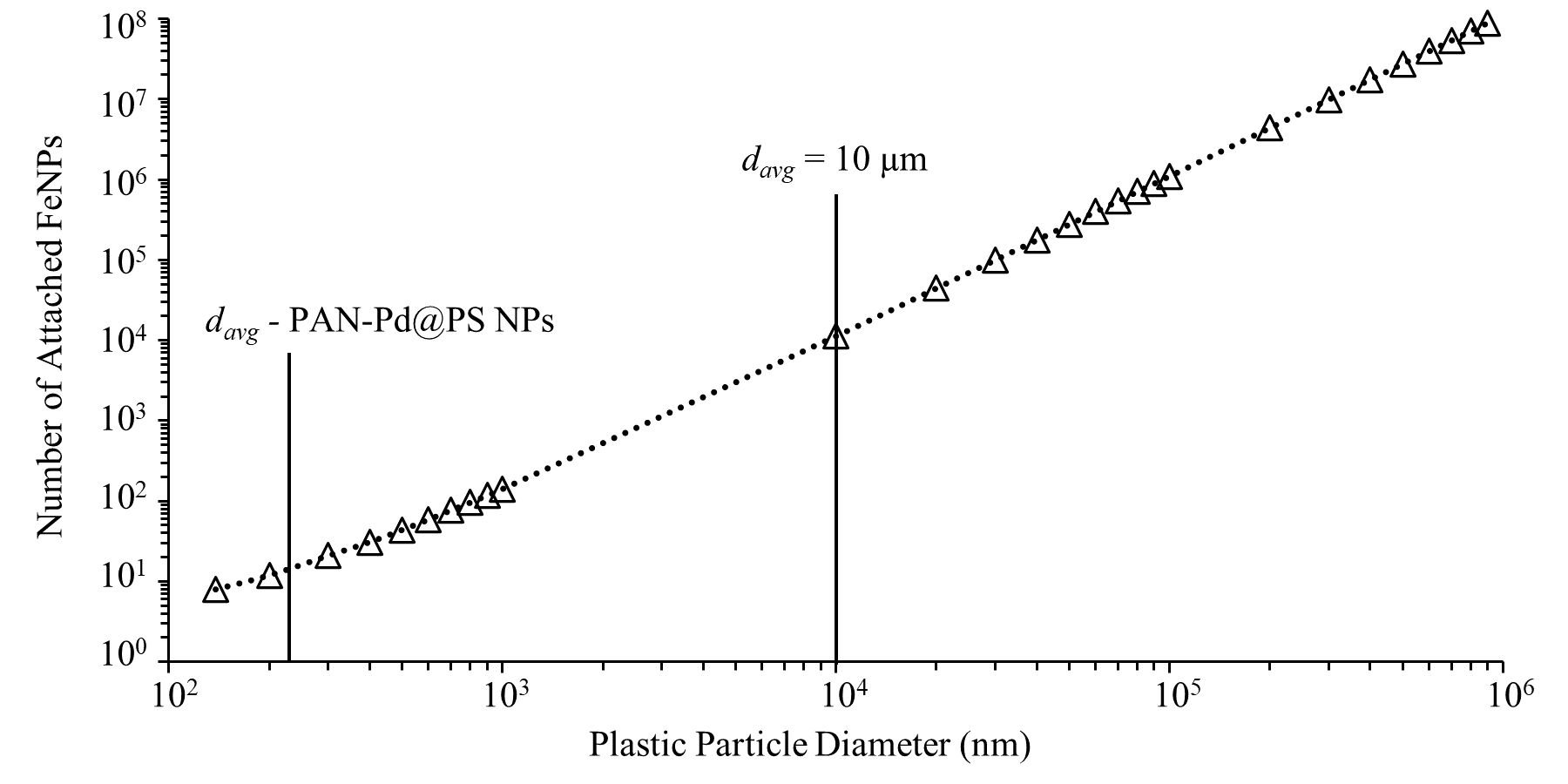


**Figure S5.** Theoretical number of HDTMS-FeNPs adsorbed to spherical plastic particles of varying size based upon Random Sequential Adsorption model. The *d_avg_* of the PAN-Pd@PS NPs measured via NTA (229 ± 1.3 nm; Table 1) is indicated.

References

(1) USEPA. Methods for Measuring the Acute Toxicity of Effluents and Receiving Waters to Freshwater and Marine Organisms Fifth Edition October 2002. **2002**, No. October, 266.

(2) Grbic, J.; Nguyen, B.; Guo, E.; You, J. B.; Sinton, D.; Rochman, C. M. Magnetic Extraction of Microplastics from Environmental Samples. *Environ. Sci. Technol. Lett.* **2019**, *6* (2), 68–72. https://doi.org/10.1021/acs.estlett.8b00671.

(3) Adamczyk, Z.; Belouschek, P. Localized Adsorption of Particles on Spherical and Cylindrical Interfaces. *J. Colloid Interface Sci.* **1991**, *146* (1), 123–136. https://doi.org/10.1016/0021-9797(91)90010-6.

(4) Sadowska, M.; Adamczyk, Z.; Nattich-Rak, M. Mechanism of Nanoparticle Deposition on Polystyrene Latex Particles. *Langmuir* **2014**, *30* (3), 692–699. https://doi.org/1ws.

(5) Svoboda, J. Magnetic Techniques for the Treatment of Materials. **2004**, 642.

(6) Rhein, F.; Scholl, F.; Nirschl, H. Magnetic Seeded Filtration for the Separation of Fine Polymer Particles from Dilute Suspensions: Microplastics. *Chem. Eng. Sci.* **2019**, *207*, 1278–1287. https://doi.org/10.1016/j.ces.2019.07.052.
